# Editorial: Combinatorial Approaches for Cancer Treatment: From Basic to Translational Research

**DOI:** 10.3389/fonc.2022.842114

**Published:** 2022-02-02

**Authors:** Aniello Cerrato, George Mattheolabakis, Daniela Spano

**Affiliations:** ^1^ Institute Experimental Endocrinology and Oncology “Gaetano Salvatore”, National Research Council, Naples, Italy; ^2^ School of Basic Pharmaceutical and Toxicological Sciences, College of Pharmacy, University of Louisiana Monroe, Monroe, LA, United States; ^3^ Institute of Biochemistry and Cell Biology, National Research Council, Naples, Italy

**Keywords:** cancer resistance, molecular mechanisms, tumor cells heterogeneity, combinatorial therapy, treatment toxicity, computational studies, preclinical and clinical studies

Cancer represents an important public health concern as the main leading cause of death worldwide. Current monotherapies, including radiotherapy, chemotherapy and molecular targeted therapy, show limited efficacy due to rapidly emerging resistance as a result either of target mutations, or engagement of parallel oncogenic pathways or onset of adaptive survival mechanisms. Moreover, the complexity of signaling networks and the activation of bypass pathway(s) render molecular targeted therapies ineffective. Therefore, combinatorial strategies could be more effective therapeutic approaches.

The most solid malignancies are characterized by hypoxia, which plays a relevant role in cancer resistance to conventional treatments (1). Therefore, therapies targeting tumor hypoxia have attracted considerable attention. Li et al. evaluate the development of prodrugs based on targeting hypoxia. They outline existing hypoxia-activated products and analyze their potential benefits in cancer treatment along with their effects in combination setting with traditional chemotherapeutics. In their second paper (Li et al.) go into detail for the hypoxia-activated prodrug TH-302 and its potential for combinatorial regime with other anti-cancer treatments, including radiotherapy, immunotherapies, anti-angiogenic agents and tissue oxygen modulators.

Oxidative stress also plays a relevant role in cancer cell sensitivity to drugs by promoting autophagy, apoptosis, or necrosis (2, 3). Zhu et al. report that metformin, an agent that exerts broad anti-cancer effects through activating AMPK and inhibiting mTOR, enhances the cytotoxicity of anlotinib, a multi-targeted tyrosine kinase inhibitor. Metformin enhances the effect of anlotinib on NADP^+^/NADPH ratio, indicating that the combination regulates intracellular redox homeostasis and promotes switching to oxidative state. The molecular mechanisms underlying the anlotinib/metformin synergistic effect involve PARP1 and caspase-3 increased cleavage and Bax/Bcl-2 enhanced ratio, suggesting that the combinatorial treatment triggers apoptosis. ROS-mediated induction of p38/JNK MAPK and ERK signaling might explain the activation of Bcl-2/Bax-caspase signaling pathway and lead to apoptosis (4).

A typical approach exploited by cancer cells to escape apoptosis is the concomitant upregulation of anti-apoptotic BCL-2 family members, including BCL-2 and MCL-1 (5). BCL-2 targeting *via* selective inhibitors ABT shows efficacy in several cancers (6, 7). However the concomitant expression of MCL-1 results in ABT-resistant cancer cells. Therefore, BCL-2/MCL-1 combined inhibition could represent a novel therapeutic approach to fight cancer. Following this hypothesis, Shi et al. investigate the therapeutic efficacy of the BCL-2 inhibitor ABT-199 combined with the MCL-1 inhibitor homoharringtonine in acute myeloid leukemia treatment. Their data suggest that the dual strategy is more effective in inducing apoptosis compared to monotherapies.

It is well known that genetic and epigenetic alterations, signaling pathways dysregulations, changes in cellular metabolism and tumor microenvironment are key players in cancer development and progression. Cancer cells metabolic reprogramming mainly consists in the activation of anaerobic glycolysis regardless of oxygen availability, which results in the higher production of glycolysis intermediates (8). Leone et al. review the dual role of Methylglyoxal, a glucose-derived reactive dicarbonyl, in cancer progression and the combined therapeutic strategies aimed to counteract tumor growth.

Lahooti et al. review the role of miRNAs in the angiogenesis. The authors present traditional anti-angiogenic chemotherapeutic agents and illustrate the potential that miRNAs have in the field. They attempt to critically evaluate how combinatorial analyses of miRNAs with traditional chemotherapeutics need to take into consideration the potential angiogenic activity of nucleic acids, an aspect frequently overlooked, even for miRNAs with known anti-angiogenic properties.

An interesting contribution is the review of Mancini et al. that raises awareness on the relevance of evaluating sex influence in preclinical and clinical trials to better comprehend sex/hormones in determining cancer development, progression, and sensitivity to therapy. Sex-related factors, mainly estrogenic hormones, affect the levels and/or function of p53 network both in hormone-dependent and -independent cancer. The review summarizes the studies reporting the relationship between sex and p53 circuitry, also focusing on preclinical studies and clinical trials to define sex effect on p53-targeted therapy. The review discusses the potential optimization of p53-targeted therapy given patients’ sex and hormonal status. Gaining insights into the effects of sex on cancer therapy can help to identify more targeted and effective combinatorial treatments.

Despite advances in the development of molecular targeted therapies, tumor heterogeneity is a relevant aspect for tumor resistance to the treatments. The chemotherapy still represents the main therapeutic approach available for treatment of some cancer types, including triple negative breast cancer (TNBC), glioblastoma, pancreatic and colorectal cancer. However, the chemotherapeutic efficacy is often limited by the occurrence of resistance whose molecular bases are still poorly understood. In the effort to identify the molecular mechanisms of carboplatin resistance in TNBC, Abreu de Oliveira et al. demonstrate that Wnt signaling activation promotes cancer stem cells (CSCs) enrichment which underlies chemotherapy resistance. Wnt signaling inhibition reduces TNBC CSCs population and resensitizes carboplatin-resistant TNBC to the drug. CSCs are key players in cancer resistance to conventional protocols of radiotherapy, chemotherapy and molecular targeted therapy, thus causing the failure of cancer therapy and tumor relapse (9). The limited clinical efficacy of monotherapeutic regimes in CSCs eradication highlights the need for developing alternative combinatorial strategies. Catara et al. review the preclinical studies which provide the proof of concept that CSCs-centered combined therapies, based on the combination of two molecular targeted therapies and the combination of molecular targeted therapy with chemotherapy or radiotherapy, result in more effective CSCs targeting compared to single treatments.

The large number of anti-cancer drugs available implies a huge number of possible combinations to be assessed to identify the synergistic ones for cancer treatment. The computational methods could represent useful tools to screen drug combinations, thus restricting the prohibitive cost and intense labor required for the experiments. Li et al. construct transcriptomics- and network-based prediction models to screen the potential drug combinations for prostate cancer treatment and evaluate their accuracy by *in vitro* assays. Their approach identifies drugs combinations to be further investigated in preclinical and clinical trials. Gondal et al. present a novel computational framework using a literature-derived *in silico Drosophila Patient Model* for treating colorectal cancer (CRC) patients. The proposed model identifies synergistic combinations for treating different CRC patients and could be deployed in preclinical settings to evaluate potential combinations before an *in vivo* evaluation. The computational approaches might help to design specific and personalized interventions taking into account the inter-individual variations occurring on genomic, biochemical, behavioral and environmental levels. Gondal and Chaudhary present a review of existing computational resources that the scientific community can utilize for evaluating existing repositories of biomolecular cancer data, in conjunction with simulation software for the development of personalized cancer therapeutics. The authors present a critical review on existing methodologies and recognize limitations in them. Crook et al. profile advanced or refractory solid cancers by multi-analyte molecular and functional tumor interrogation [Encyclopedic Tumor Analysis (ETA)] to select personalized combination treatment regimens. The ETA-guided combined treatments of mTOR inhibitors with several anti-neoplastic agents, targeting different tumor-associated signaling pathways, improve significantly the Progression Free Survival of treated patients, thus providing clinical evidence of the therapeutic benefit achieved by personalized combinatorial strategies compared to mTOR inhibitors as monotherapies.

Among the combinatorial strategies, the local treatment based on photothermal therapy (PTT) might benefit of immunotherapy in combination setting. This strategy could enhance the efficacy with a limited toxicity of the treatment. Although the immunotherapy in combination setting is the subject of other Frontiers in Oncology Research Topics, the paper of Xia et al., which combine immunotherapy and PTT (10) for local cancer treatment of hepatocarcinoma, is included in this Research Topic as strategy to potentiate the local cancer treatment. PTT induces the release of tumor-associated antigens by ablating tumor while Ganoderma Lucidum polysaccharides exert the anti-tumor action by stimulating the immune function without apparent toxicity.

In summary, this Research Topic is focused on the combinatorial treatment strategies to fight cancer which target cancer hallmarks, including signaling pathway dysregulation, hypoxia, oxidative stress, metabolic reprogramming, angiogenesis and stemness. The papers included in this Research Topic strengthen the relevance of combined approaches compared to single treatments in preclinical and in clinical setting.

## Author Contributions

All authors listed have made a substantial, direct and intellectual contribution to the work, and approved it for publication.

## Funding

This work was supported for GM by the College of Pharmacy, University of Louisiana Monroe start-up funding and the National Institutes of Health (NIH) through the National Institute of General Medical Science Grants 5 P20 GM103424-15, 3 P20 GM103424-15S1.

## Conflict of Interest

The authors declare that the research was conducted in the absence of any commercial or financial relationships that could be construed as a potential conflict of interest.

## Publisher’s Note

All claims expressed in this article are solely those of the authors and do not necessarily represent those of their affiliated organizations, or those of the publisher, the editors and the reviewers. Any product that may be evaluated in this article, or claim that may be made by its manufacturer, is not guaranteed or endorsed by the publisher.
